# Can Non-Conventional Blood Biomarkers Improve Running Performance Prediction? A Proof of Concept

**DOI:** 10.3390/life16020320

**Published:** 2026-02-12

**Authors:** Matija Dvorski, Marija Rakovac, Tomislav Kelava, Nataša Kovačić, Darja Flegar, Sara Aničić, Ivo Krešić, Ljiljana Ćulibrk, Filip Koražija, Damjan Dimnjaković, Alan Šućur

**Affiliations:** 1 Department of Environmental and Occupational Health and Sports Medicine, Andrija Stampar School of Public Health, School of Medicine, University of Zagreb, 10000 Zagreb, Croatia; matija.dvorski@snz.hr; 2 Department of Kinesiological Anthropology and Methodology, Faculty of Kinesiology, University of Zagreb, 10110 Zagreb, Croatia; 3 Laboratory for Molecular Immunology, Croatian Institute for Brain Research, University of Zagreb, 10000 Zagreb, Croatia; tomislav.kelava@mef.hr (T.K.); natasa.kovacic@mef.hr (N.K.); darja.flegar@mef.hr (D.F.); sara.anicic@mef.hr (S.A.); ivo-kresic@mefhr.org (I.K.); alan.sucur@mef.hr (A.Š.); 4 Department of Physiology and Immunology, School of Medicine, University of Zagreb, 10000 Zagreb, Croatia; 5 Department of Anatomy, School of Medicine, University of Zagreb, 10000 Zagreb, Croatia; 6 Department of Physiology, School of Medicine, University of Mostar, 88000 Mostar, Bosnia and Herzegovina; 7 Athletic Club Agram, 10000 Zagreb, Croatia; agram@atletskiklubagram.hr; 8 Running Club and Academy Trčaona, 10000 Zagreb, Croatia; filip@aktivan-zivot.hr; 9 Department of Orthopaedic Surgery, University Hospital Centre Zagreb, 10000 Zagreb, Croatia; damjan.dimnjakovic@mef.hr

**Keywords:** blood-based biomarkers, brain-derived neurotrophic factor, cardiorespiratory fitness, decorin, hypoxanthine, prediction model, running performance, N-terminal pro-B-type natriuretic peptide

## Abstract

Conventional measures such as maximal oxygen uptake ($\overset{˙}{V}$O_2_max), although widely regarded as the gold standard, do not fully capture endurance performance. Therefore, this study investigated whether a 2.4 km Cooper test elicits measurable changes in blood-based biomarkers (decorin, hypoxanthine, N-terminal pro-B-type natriuretic peptide (NT-proBNP), brain-derived neurotrophic factor (BDNF)) and whether integrating these markers may improve performance prediction in a heterogeneous sample of runners. In this cross-sectional observational proof-of-concept study, thirty-three participants completed the 2.4 km Cooper test, with venous blood samples collected at baseline and post-test. Non-parametric statistical tests were used to assess biomarker changes (*α* = 0.05), with exploratory correlations evaluated using Spearman’s *ρ*. To examine whether blood-based biomarkers provide information beyond conventional field-based predictors, Ridge regression with leave-one-out cross-validation (LOOCV) was applied to predict 10 km race time in a subsample of 24 participants who completed a 10 km race two weeks later. The Cooper test elicited significant post-test changes in decorin, hypoxanthine, and BDNF (all *p* < 0.001). Higher post-test decorin (*ρ* = −0.44, *p* = 0.010) and hypoxanthine (*ρ* = −0.37, *p* = 0.034) were associated with faster Cooper test performance. In Ridge regression analysis, adding post-test decorin to conventional predictors resulted in a minor reduction of 10 km race time prediction error. This study suggests that decorin may provide complementary information to a conventional field-based test in heterogeneous recreational runners. Post-test decorin marginally contributed to 10 km race performance prediction beyond established predictors, though external validation and comparison with directly measured $\overset{˙}{V}$O_2_max are needed before practical application can be recommended.

## 1. Introduction

Accurate performance prediction is fundamental to optimizing training prescription and competitive strategy in endurance sports [1,2]. However, an unresolved challenge remains: traditional field tests, while practical, often lack sufficient sensitivity to capture the physiological nuances that differentiate performance levels within heterogeneous recreational cohorts [3]. While these tests provide a broad estimate of capacity, they frequently fail to provide the incremental predictive value necessary for precise individualized monitoring.

Maximal oxygen uptake ($\overset{˙}{V}$O_2_max) is widely regarded as the reference measure for assessing aerobic capacity and a primary predictor of endurance performance [4]. However, direct laboratory measurement is often impractical, making field-based alternatives essential [5]. The 2.4 km Cooper test is among the most valid and appropriate field tests for estimating $\overset{˙}{V}$O_2_max across sex, age, and fitness-level subgroups, showing strong criterion-related validity (pooled *r*_p_ ≈ 0.79) [6]. As a maximal effort test requiring athletes to cover the distance as quickly as possible, it also captures elements of pacing and mental endurance that are relevant to competitive racing [7,8]. However, the predictive accuracy of such field tests is often constrained by variability in pacing strategies, individual motivation, and environmental conditions, which can decouple estimated capacity from actual race potential [9]. This is particularly relevant in recreational runners, where these confounding factors are more pronounced than in elite populations. Consequently, the 2.4 km Cooper test may serve as a useful, albeit imperfect, proxy that warrants supplementary physiological data to improve its predictive precision [10].

Nevertheless, $\overset{˙}{V}$O_2_max alone does not reliably predict performance, particularly among athletes with comparable aerobic capacity [11]. Conventional markers such as creatine kinase (CK) for muscle damage, hormonal ratios (e.g., testosterone-to-cortisol ratio (TCR)) for metabolic balance, the neutrophil-to-lymphocyte ratio (NLR) for systemic inflammation, and lactate are widely used but have significant limitations [12,13]. Their utility is often constrained by high variability, delayed responses, and sensitivity to external factors [14]. Lactate, for instance, responds immediately, but a single measurement primarily captures acute exercise intensity because it reflects the net balance between production and removal. Therefore, it is less indicative of underlying training adaptations than graded protocols [15,16]. This ambiguity highlights the need for novel biomarkers that can provide a more comprehensive evaluation of an athlete’s training status and competitive potential [12].

To address this, we selected biomarkers representing distinct physiological domains not captured by conventional metabolic thresholds. While lactate and ventilatory thresholds provide valuable metabolic information, they primarily reflect oxidative-glycolytic balance and cannot distinguish between musculoskeletal robustness, cardiac stress, or neuroplastic adaptation [16].

Therefore, we chose four biomarkers with non-overlapping physiological provenance: decorin, hypoxanthine, N-terminal pro-B-type natriuretic peptide (NT-proBNP), and brain-derived neurotrophic factor (BDNF). Hypoxanthine reflects purine metabolism and adenosine triphosphate (ATP) degradation; decorin is involved in skeletal muscle extracellular matrix (ECM) remodeling; NT-proBNP indicates cardiac wall stress; and BDNF reflects central nervous system neuroplasticity.

This approach aimed to identify which physiological domain, if any, provides information beyond conventional aerobic capacity measures.

Decorin, a small leucine-rich proteoglycan that also functions as a myokine, is secreted in response to muscle contraction [17]. It promotes muscle hypertrophy and ECM remodeling by suppressing myostatin activity and is influenced by growth hormone signaling, with stronger exercise-induced increases reported in men [17,18,19]. Because the ECM is critical for the storage and return of elastic energy during the gait cycle, the magnitude of the acute post-exercise decorin response may reflect functional properties of the muscle–tendon unit (e.g., ECM organization and muscle–tendon stiffness) that are relevant for running economy and, consequently, for 10 km performance [20].

Hypoxanthine is a byproduct of ATP degradation in skeletal muscle. Its plasma concentration increases several-fold after intense exercise, reflecting acute metabolic stress [21]. As hypoxanthine response depends on the acute metabolic load of exercise, well-trained individuals may demonstrate higher hypoxanthine accumulation after maximal or near-maximal efforts because of their greater absolute metabolic power [21,22]. At the same time, lower post-exercise levels often indicate enhanced purine salvage capacity in trained athletes, making it a potential indicator of both metabolic stress and training status [22,23].

NT-proBNP is secreted in response to cardiac ventricular wall stress. While endurance exercise transiently elevates its levels, well-trained athletes typically exhibit lower resting concentrations, reflecting positive myocardial adaptations [24].

BDNF is a neurotrophin that is essential for synaptic plasticity. It crosses the blood–brain barrier, and its peripheral concentrations correlate with central nervous system levels [25]. Its circulating levels increase significantly with acute aerobic exercise [26,27]. Resting BDNF has been negatively correlated with $\overset{˙}{V}$O_2_max, suggesting a role in neuroplastic adaptations to training [27].

Previous research on these biomarkers is fragmented, focusing on single markers with heterogeneous study designs and lacking integration with performance measures [14]. Specifically, there is a methodological gap regarding whether these non-conventional markers provide incremental predictive value beyond established field-based predictors. This lack of applied validation makes it difficult to determine if biomarker monitoring can practically refine performance estimations in a real-world racing context. This study aimed to overcome these limitations by simultaneously measuring all four biomarkers in response to a single, standardized field test.

The selection of these biomarkers was guided by previous studies indicating their responsiveness to high-intensity aerobic exercise protocols with metabolic and neuromuscular demands comparable to those of the Cooper test [17,23,27,28]. We hypothesized that incorporating non-conventional biomarkers, specifically their acute post-test concentrations or their absolute changes, into a prediction model would provide incremental predictive value for 10 km race time. While prior research has largely been descriptive—documenting the responsiveness of biomarkers to exercise—this study evaluates their incremental predictive value. Specifically, we evaluated whether these markers could provide additive information for performance forecasting that is not already captured by conventional field-based predictors.

## 2. Materials and Methods

### 2.1. Research Design

This study was a cross-sectional observational proof-of-concept study with an acute exercise challenge. The required sample size was estimated a priori using G*Power (version 3.1.9.6; Heinrich Heine University Düsseldorf, Germany) for a two-tailed paired-samples *t*-test to detect a medium effect size (*d* = 0.5) for changes in biomarker concentrations. The type I error rate was set to *α* = 0.05, with statistical power of 0.80.

Field testing was conducted on a single day on an outdoor 400 m athletic track between 8:00 and 10:00 a.m., under stable environmental conditions. Environmental data were obtained from hourly meteorological records for the testing location during the testing window (mean ± standard deviation values for temperature: 11.9 ± 0.4 °C; relative humidity: 85 ± 8.5%; atmospheric pressure: 1021.9 ± 0.8 hPa; wind speed: 2.5 ± 0.7 m/s) [29].

### 2.2. Participants

Thirty-three runners (22 male, 11 female) from Zagreb athletics clubs, representing sprint (*n* = 3), middle-distance (*n* = 3), and long-distance disciplines (*n* = 27), were recruited on a volunteer basis via announcements distributed by club coaches. The heterogeneity of the cohort aimed to increase the generalizability of the biomarkers across a spectrum of recreational runners. This likely resulted in greater between-subject variability in acute biomarker responses and in the observed strength of biomarker–performance associations.

The inclusion criteria required participants to be 18–35 years old, train at least twice per week, and have completed at least one race of 2.4 km or longer within the previous two years. Individuals with a history of chronic disease or use of alcohol, tobacco, medication, or psychoactive substances within the last six months were excluded. To ensure standardized conditions, all participants were instructed to avoid high-intensity exercise and high-salt or high-fat meals for 48 h prior to testing. Additionally, they were required to maintain normal hydration and abstain from food and caffeine for at least three hours before their scheduled arrival. Only healthy, consenting individuals who adhered to these pre-test instructions were enrolled on the day of testing.

The study was conducted in accordance with the Declaration of Helsinki and approved by the Ethics Committee of the University of Zagreb School of Medicine (approval number 251-59-10106-24-111/126). All participants and participating clubs provided written informed consent.

### 2.3. Experimental Protocol and Performance Measures

Upon arrival and prior to exercise, demographic data (age, sex), training history, and anthropometric measurements were collected. Body mass (to 0.1 kg) and body composition (fat percentage, muscle mass) were assessed via single-frequency (50 kHz) segmental bioelectrical impedance analysis (Tanita BC-418MA; TANITA Corp., Tokyo, Japan) using the athletic mode after bladder voiding, with a standardized 0.5 kg clothing mass subtracted [30]. Body height was measured to 1 cm using an anthropometer (GPM Instruments GmbH, Susten, Switzerland). Data were processed with GMON Pro software (version 3.4.8; Medizin & Service GmbH, Chemnitz, Germany), and body mass index (BMI) was calculated.

Following these baseline measurements, participants performed a standardized 15 min warm-up of light jogging and dynamic stretching [31,32]. They then completed a 2.4 km Cooper test at maximal effort, with completion time recorded to 0.01 s (Terinda Stopwatch Professional; Terinda d.o.o., Slovenska Bistrica, Slovenia). Immediately post-test, participants rated their perceived exertion (RPE) on the Borg CR-10 scale [33]. The primary performance outcome, 10 km race time, was obtained two weeks later from a subsample of 24 participants who competed in an official road race. These official results, obtained from the event organizer’s website [34], served as the criterion variable for performance prediction.

### 2.4. Blood Collection and Laboratory Analyses

Venous blood samples were collected by qualified medical staff at two time points: pre-warm-up in a fasting state (6 mL) and within 10 min of completing the Cooper test (12 mL). The sampling window was selected in accordance with previous studies [17,23,27,28] which showed measurable increase in chosen markers after comparable physical activity, while also ensuring logistical feasibility in a field setting. Blood was drawn into EDTA Vacutainer tubes for plasma and clot activator tubes for serum (Becton Dickinson; Franklin Lakes, NJ, USA). Serum tubes were rested for 30–60 min for clot formation before all samples were centrifuged within two hours. Plasma (1000× *g* for 20 min) and serum (1000× *g* for 10 min) aliquots were stored at −80 °C for three weeks until analysis.

Hypoxanthine was measured in deproteinized plasma using a fluorescence-based assay kit (Xanthine/Hypoxanthine Assay Kit, ab155900; Abcam, Cambridge, UK). Decorin (Human Decorin ELISA Kit, ab99998), BDNF (Human BDNF ELISA Kit, ab212166), and NT-proBNP (Human NT-proBNP ELISA Kit, ab263877) were measured in serum using commercial enzyme-linked immunosorbent assay (ELISA) kits from Abcam (Cambridge, UK). All assays were performed according to the manufacturer’s instructions. Absorbance and fluorescence were measured on an Infinite^®^ 200 Pro microplate reader (Tecan Group Ltd., Männedorf, Switzerland). Key analytical characteristics of the assays (intra- and inter-assay coefficients of variation and minimum detectable doses) are provided in Supplementary Table S1.

A panel of conventional markers, including complete blood count (CBC), CK, LDH, testosterone, and cortisol, was analyzed from the post-test sample only. CBC was determined from whole blood using an ADVIA^®^ 2120i automated hematology analyzer (Siemens Healthineers, Erlangen, Germany). Post-test serum concentrations of testosterone and cortisol were determined via immunoassay on an Immulite^®^ 2000 XPi analyzer (Siemens Healthineers, Erlangen, Germany), while CK and LDH were measured using a BA400 biochemical analyzer (BioSystems, Barcelona, Spain).

### 2.5. Data Processing and Statistical Analysis

Estimated maximal oxygen uptake ($\overset{˙}{V}$O_2_max, mL kg^−1^ min^−1^) was calculated from Cooper test time, sex, and body mass using a validated equation [32]. Predicted 10 km running time was calculated using Riegel’s formula with a fatigue factor of 1.06 [35]. For statistical analysis, NT-proBNP values below the lower limit of detection (LoD) were imputed as half of the LoD.

All statistical analyses were performed using IBM SPSS Statistics for Windows (version 27.0; IBM Corp., Armonk, NY, USA), with regularized regression analyses implemented in Python (version 3.12.12; Python Software Foundation, Wilmington, DE, USA) with the scikit-learn library (version 1.6.1) [36]. Data distribution was assessed with the Shapiro–Wilk test. Non-parametric tests were used for comparisons: the Wilcoxon signed-rank test for within-group changes and the Mann–Whitney *U* test for between-group differences. Effect sizes were calculated as *r* = *z*/$\sqrt{n}$ [37]. Exploratory correlation analyses were performed using Spearman’s (*ρ*) coefficients, and analysis of covariance (ANCOVA) was used to control for covariates. Scatterplots were examined prior to correlation analysis. Statistical significance was set at *α* = 0.05. No formal correction for multiple testing was applied.

To classify participants by training status, k-means cluster analysis was performed on the standardized *z*-scores of four variables with low intercorrelation (|*ρ*| < 0.80): estimated $\overset{˙}{V}$O_2_max, average weekly running volume, years of running experience, and body fat percentage. The number of clusters was predetermined as two (higher and lower training status).

To predict 10 km performance (race), regularized regression models were developed, avoiding the inclusion of highly correlated predictors (|*ρ*| > 0.80) in the same model. Given the small sample size and the hypothesis that multiple biomarkers contribute additively to performance, Ridge regression (L2 penalization) was selected as the primary method. This was based on Ridge’s penalty term that shrinks coefficient estimates towards zero, thereby stabilizing the model and reducing the risk of overfitting inherent in small datasets with correlated predictors. Elastic Net (L1/L2 penalization) was subsequently used as a robustness check [38]. All predictor variables were standardized with StandardScaler within the training folds, and hyperparameters were tuned using GridSearchCV with k-fold cross-validation. For Ridge regression, the hyperparameter *α* (regularization strength) was tuned in the range 0.01–1.00 (step 0.05). For Elastic Net, both *α* (0.01–1.00, step 0.05) and the L1 ratio (0.01–1.00, step 0.10) were tuned.

For in-sample analyses, hyperparameters were selected once using 6-fold cross-validation on the full dataset and then fixed for model fitting and reporting of in-sample performance metrics. All models were developed using data from the 24 participants who completed the 10 km race. Predictive performance was assessed using leave-one-out cross-validation (LOOCV) to obtain an internally evaluated estimate of predictive performance. For LOOCV, a nested cross-validation procedure was applied. In each leave-one-out iteration, the held-out participant was excluded. Model hyperparameters were re-tuned using 5-fold cross-validation on the remaining *n* − 1 participants. Five folds were used to ensure sufficiently sized folds in the reduced training set. The resulting model was then used to generate the held-out prediction.

Evaluation included the coefficient of determination (*R*^2^), mean absolute error (MAE), and root-mean-square error (RMSE) for race time predictions.

Bootstrap 95% BCa CIs (2000 resamples) were computed for regression coefficients to assess the stability of coefficient estimates in the small sample. Ridge regression coefficients are reported descriptively rather than inferentially because penalization introduces bias in coefficient estimates in small samples [38].

## 3. Results

### 3.1. Participant Characteristics and Performance Metrics

The study population (*n* = 33; 22 males, 11 females) had a median age of 25 years (range: 18–33) and a median BMI of 20.9 kg m^−2^. The median body fat percentage was 14.1%. These data are shown in Table 1 (see also Supplementary Table S2 for sex-based comparisons). The cohort was heterogeneous in training background; their average weekly running volume ranged from <20 km to >100 km, with the largest proportion of participants (42.4%) reporting 20–39 km. Running experience extended up to ≥10 years, with most participants (30.3%) reporting 1–2 years.

Participants completed the 2.4 km Cooper test with times ranging from 7.11 to 13.48 min, corresponding to estimated $\overset{˙}{V}$O_2_max values of 39.0 to 61.7 mL kg^−1^ min^−1^. The exertion was perceived as near-maximal (median RPE = 9). In the subsample that competed in the 10 km race (*n* = 24), official finish times ranged from 31.73 to 61.40 min (Table 2). Participants who did not participate in the 10 km race (*n* = 9) did not differ from race completers in Cooper test performance, anthropometric characteristics, body composition, average weekly training volume, or running experience (all *p* > 0.05).

Based on $\overset{˙}{V}$O_2_max, average weekly running volume, experience, and body fat percentage, k-means clustering stratified the cohort into higher training status (HTS, *n* = 19) and lower training status (LTS, *n* = 14) groups. All included variables significantly differentiated the two groups (all *p* < 0.01).

The HTS group was significantly younger and had a lower body fat percentage than the LTS group (Supplementary Table S3), while demonstrating superior performance in both the Cooper test (mean: 8.18 vs. 10.89 min) and the 10 km race (mean: 36.80 vs. 50.74 min; both *p* < 0.001). HTS participants also reported substantially greater average weekly running volumes, with 63.2% exceeding 60 km. In contrast, the majority of LTS participants (71.4%) reported average weekly running volumes of 20–39 km.

### 3.2. The 2.4 km Cooper Test Elicits Robust Biomarker Changes

**Figure 1:**
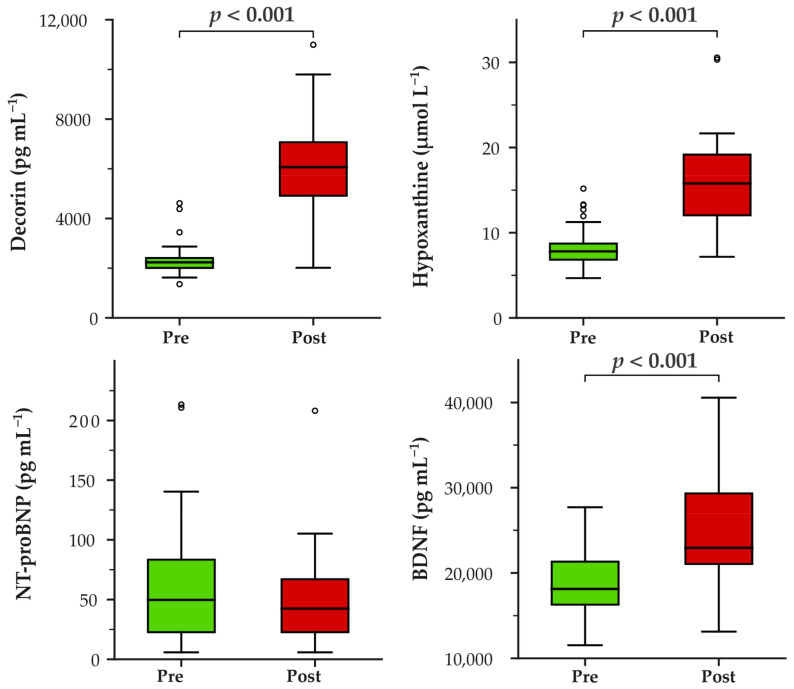
Blood-based biomarker changes after the 2.4 km Cooper test. Concentrations of decorin, hypoxanthine, NT-proBNP, and BDNF measured at baseline (Pre) and after (Post) the 2.4 km Cooper test in the study population (*n* = 33). Red boxes represent post-test values, and green boxes represent baseline values. Differences between Pre and Post were tested using the Wilcoxon signed-rank test. Statistically significant differences (*p* < 0.05) are indicated above the boxes. The central line within the box indicates the median; whiskers denote 1.5 × interquartile range; circles indicate outliers. NT-proBNP—N-terminal pro-B-type natriuretic peptide; BDNF—brain-derived neurotrophic factor.

The 2.4 km Cooper test evoked significant changes in three of the four biomarkers. Post-test concentrations of decorin, hypoxanthine, and BDNF were significantly higher than baseline (all *p* < 0.001), with large effect sizes (*r* = 0.80–0.87). In contrast, NT-proBNP did not change significantly (*p* = 0.185) (Figure 1).

When analyzed by subgroups, these increases were observed in both sexes and in both HTS and LTS participants (all *p* < 0.01; *r* = 0.71–0.88). While post-test decorin was higher in males (Supplementary Figure S1a), the effect of sex was no longer significant after controlling for muscle mass, which showed a strong independent effect (*F*_1,31_ = 27.92; *p* < 0.001; *η*^2^_p_ = 0.47). Similarly, comparing by training status, no significant baseline differences were found. However, post-test hypoxanthine was significantly higher in the HTS group compared to LTS (*p* = 0.038) (Supplementary Figure S1b).

### 3.3. Correlation Between Blood-Based Biomarkers and Running Performance

#### 3.3.1. Correlation with 2.4 km Cooper Test Time

**Figure 2:**
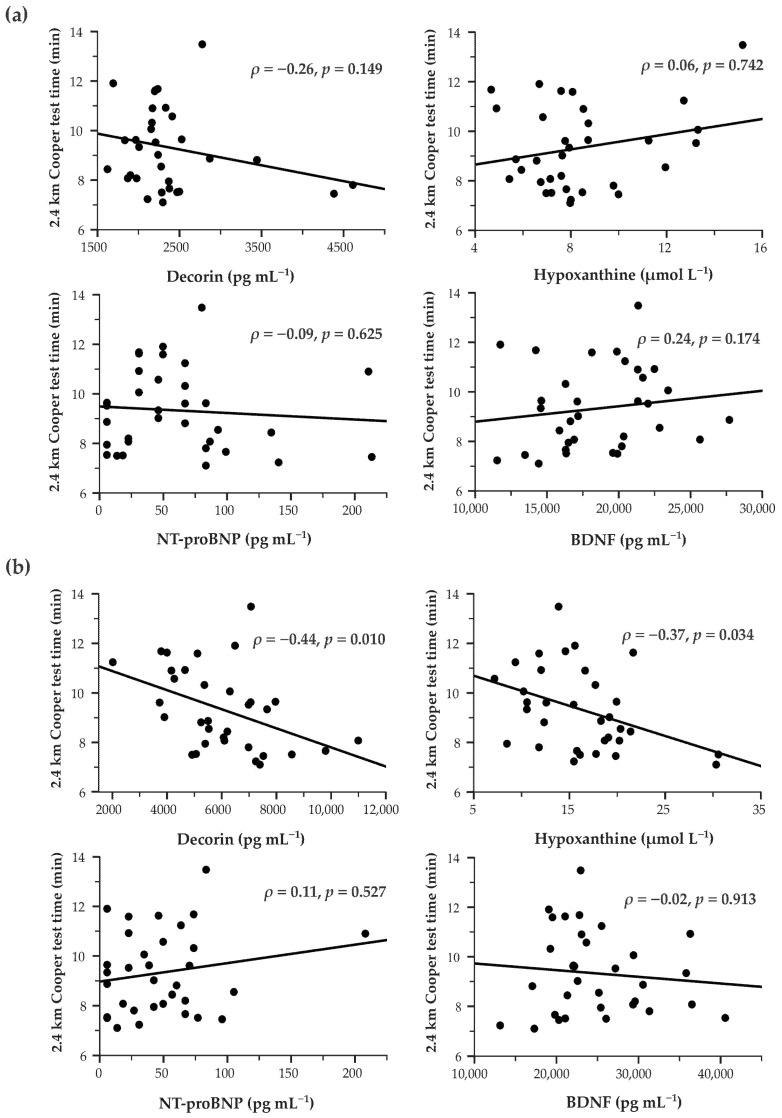
Correlations between blood-based biomarkers and 2.4 km Cooper test running time. Scatterplots showing correlations between (**a**) baseline and (**b**) post-test biomarker concentrations and 2.4 km Cooper test running time in the study population (*n* = 33). Solid lines represent linear trendlines. Correlation coefficients were computed using Spearman’s *ρ*. Statistically significant correlation was determined at *p* < 0.05. NT-proBNP—N-terminal pro-B-type natriuretic peptide; BDNF—brain-derived neurotrophic factor.

Baseline biomarker concentrations were not significantly correlated with Cooper test time (all *p* > 0.05) (Figure 2a). However, higher post-test decorin (*ρ* = −0.44, *p* = 0.010) and hypoxanthine (*ρ* = −0.37, *p* = 0.034) were significantly correlated with faster performance (Figure 2b). When stratified by training status, these correlations showed non-significant trends (Supplementary Figure S2a,b).

The absolute change (Δ = post − pre) in hypoxanthine and decorin was also significantly negatively correlated with Cooper test time (*ρ* = −0.37, *p* = 0.036; *ρ* = −0.36, *p* = 0.037, respectively). Additionally, baseline hypoxanthine was negatively correlated with its absolute change (*ρ* = −0.48, *p* = 0.005), indicating smaller increases in those with higher starting levels.

#### 3.3.2. Correlation with 10 km Race Time

**Figure 3:**
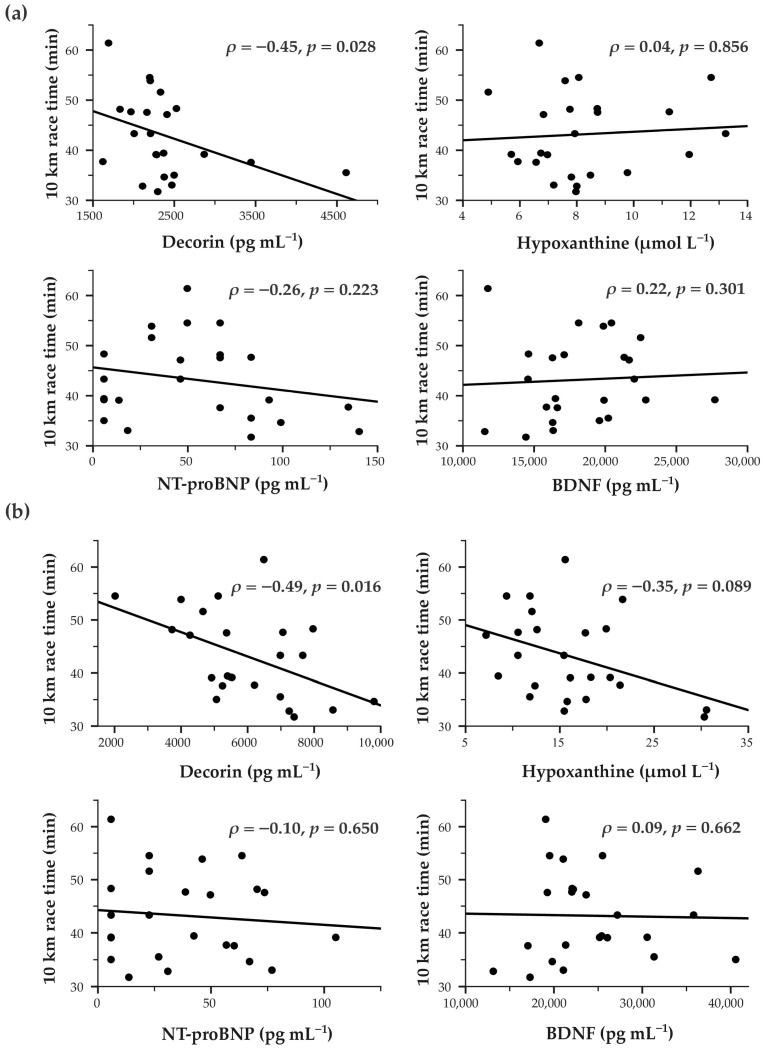
Correlations between blood-based biomarkers and 10 km road race time. Scatterplots showing correlations between (**a**) baseline and (**b**) post-test biomarker concentrations (measured in relation to the 2.4 km Cooper test) and 10 km road race time in the study population (*n* = 24). Solid lines represent linear trendlines. Correlation coefficients were computed using Spearman’s *ρ*. Statistically significant correlation was determined at *p* < 0.05. NT-proBNP—N-terminal pro-B-type natriuretic peptide; BDNF—brain-derived neurotrophic factor.

Both baseline (*ρ* = −0.45, *p* = 0.028) and post-test (*ρ* = −0.49, *p* = 0.016) decorin were significantly correlated with faster 10 km race times (Figure 3). Post-test hypoxanthine, NT-proBNP and BDNF showed no significant correlations. In a stratified analysis, a significant positive correlation between baseline BDNF and race time was present only in the HTS group (*ρ* = 0.71, *p* = 0.006) (Supplementary Figure S3).

Overall, inspection of the scatterplots suggested that the reported significant biomarker–performance associations were not dominated by single extreme values.

### 3.4. Correlation with Conventional Blood-Based Markers

**Figure 4:**
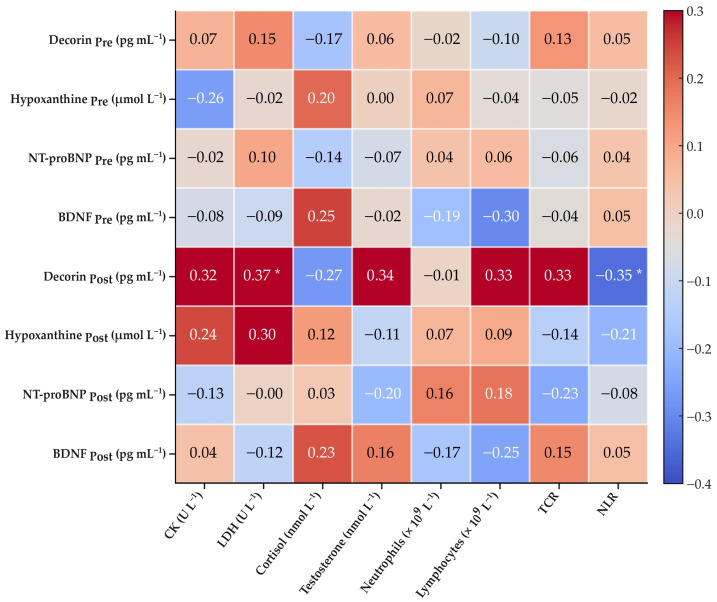
Correlations between non-conventional and conventional markers. Heatmap showing correlations between non-conventional blood-based biomarkers (decorin, hypoxanthine, NT-proBNP, and BDNF) measured at baseline (Pre) and after (Post) the 2.4 km Cooper test, and conventional blood markers measured after the test in the study population (*n* = 33). Numbers represent correlation coefficients computed using Spearman’s *ρ*. Asterisks (*) indicate significant correlations (*p* < 0.05). NT-proBNP—N-terminal pro-B-type natriuretic peptide; BDNF—brain-derived neurotrophic factor; CK—creatine kinase; LDH—lactate dehydrogenase; TCR—testosterone-to-cortisol ratio; NLR—neutrophil-to-lymphocyte ratio.

In the overall population, post-test decorin was positively correlated with LDH (*ρ* = 0.37, *p* < 0.05) and negatively correlated with the NLR (*ρ* = −0.35, *p* < 0.05) (Figure 4). When stratified by training status, baseline decorin was negatively correlated with CK (*ρ* = −0.51, *p* < 0.05), and post-test decorin was negatively correlated with the NLR (*ρ* = −0.55, *p* < 0.05) in the HTS group (Supplementary Figure S4).

### 3.5. Race Time Prediction Model

A sequential approach was applied to develop a Ridge regression model for predicting 10 km race time. Predictor inclusion was guided by three criteria: (1) biological plausibility, (2) consistent improvement in MAE and RMSE under both in-sample and LOOCV evaluation, with consideration of changes in *R*^2^, and (3) achieving a parsimonious final model.

First, a model using only the non-conventional blood-based biomarkers was tested. NT-proBNP was not considered as a predictor due to the absence of significant changes after the Cooper test. Among the remaining biomarkers (decorin, hypoxanthine, and BDNF) and their pre-test, post-test, absolute, and relative change values, post-test decorin explained the most variance in 10 km race time within this sample, albeit with a low coefficient of determination (*R*^2^ = 0.235) and a relatively large prediction error (MAE = 5.78 min).

Because standalone decorin had limited predictive strength, we evaluated both Riegel-predicted 10 km race time and estimated $\overset{˙}{V}$O_2_max as baseline predictors, given their established roles in endurance performance prediction [4,35]. In this sample, they showed comparable model fit (*R*^2^ ≈ 0.92), with the Riegel-predicted time yielding a slightly lower error (MAE = 1.755 min) than the estimated $\overset{˙}{V}$O_2_max (MAE = 1.762 min). Given the high predictive strength of this baseline model, subsequent extensions were evaluated primarily based on their ability to reduce prediction error.

As a second step, anthropometric and body composition variables (Table 1) were evaluated to assess whether model performance could be improved by incorporating routinely measured physiological characteristics. BMI provided the largest improvement when included in the Riegel-based model, reducing the MAE from 1.76 to 1.56 min (≈11%) compared with the Riegel-predicted time alone. To avoid collinearity, anthropometric and body composition variables were not combined with $\overset{˙}{V}$O_2_max, as the latter is calculated using a formula that already includes body mass [32]. Conventional biomarkers (Supplementary Table S4) were not included, to maintain model parsimony.

As a third step, we evaluated whether including blood-based biomarker pre-test, post-test, absolute, or relative change values could further improve prediction performance. Among all evaluated parameters, only post-test decorin provided a consistent and LOOCV-stable improvement. It reduced in-sample MAE by an additional 0.19 min and yielded a total reduction of 0.39 min (≈22%) relative to the Riegel-only model. With its inclusion, the model showed improved in-sample fit, accounting for approximately 96% of the variance in 10 km race time.

The sequential improvement in 10 km race time prediction using Ridge regression is summarized in Table 3. Agreement between observed and LOOCV-predicted 10 km race times for the final three-predictor model is illustrated in Supplementary Figure S5. The standardized and unstandardized coefficients for this three-predictor model are shown in Table 4. Bootstrap 95% BCa confidence intervals indicate stable standardized coefficient estimates, whereas the unstandardized decorin_post_ (ng mL^−1^) coefficient estimate shows greater uncertainty. For regression analyses, decorin concentrations were rescaled from pg mL^−1^ to ng mL^−1^ solely to facilitate the interpretation and presentation of model coefficients.

Elastic Net regression (*α* = 0.01, L1 ratio = 0.31), applied to the final three-predictor model, performed comparably to the Ridge model in both in-sample (*R*^2^ = 0.955; MAE = 1.37 min, RMSE = 1.70 min) and LOOCV evaluation (*R*^2^ = 0.931; MAE = 1.75 min, RMSE = 2.10 min), indicating that introducing an L1 component did not improve cross-validated predictive performance, and supporting the robustness of the three-predictor Ridge solution.

## 4. Discussion

The central finding of our study is twofold. First, the 2.4 km Cooper test is a valid and practical protocol for eliciting acute musculoskeletal (decorin), metabolic (hypoxanthine), and neurotrophic (BDNF) responses in a heterogeneous sample of recreational runners. Second, among the four biomarkers examined, only post-test decorin contributed additional predictive information for 10 km race time beyond established field-based predictors (Riegel-predicted time and BMI). This improvement was consistent across multiple analytical approaches (Ridge and Elastic Net regression; in-sample and LOOCV evaluation), though its absolute magnitude was small.

### 4.1. Biomarker Changes in the Context of Existing Research

#### 4.1.1. Decorin as an Indicator of Musculoskeletal Robustness

Higher post-test decorin was significantly correlated with better performance in both the 2.4 km and 10 km races. While unadjusted post-test decorin concentrations were higher in males—consistent with sex-dimorphic growth hormone responses [19]—this disparity was not significant after controlling for muscle mass. This suggests that decorin’s release reflects the volume of activated contractile tissue, rather than biological sex per se. Accordingly, the coefficient for post-test decorin in our model might capture a musculoskeletal robustness factor that is mechanistically applicable to both sexes, though its minor predictive strength would position it as a complementary, rather than a standalone, predictor.

This finding is consistent with decorin’s role as a myokine involved in extracellular matrix (ECM) remodeling, muscle growth, and mechanical loading responses [17,19,39,40]. As a critical component of the skeletal muscle ECM, decorin regulates collagen fibrillogenesis to enhance tissue integrity and inhibits myostatin, a potent negative regulator of muscle growth, thereby fostering an anabolic environment for muscle repair and adaptation [41]. A robust post-exercise decorin response may be associated with ‘musculoskeletal robustness’—an enhanced ability to sense mechanical load and initiate an efficient adaptive cascade. This quality could manifest as superior running economy, as a well-organized ECM is critical for the storage and return of elastic energy during each stride [42].

This interpretation is supported by a recent study where post-race decorin in marathon runners was correlated positively with $\overset{˙}{V}$O_2_max [43]. Our observation of an inverse correlation between baseline decorin and post-test CK in HTS runners aligned with these findings, suggesting a protective role in maintaining muscle integrity under repetitive loading [39]. It is notable that elevated baseline decorin concentrations were significantly correlated with faster 10 km race times. This suggests that circulating decorin may reflect not only an acute secretory response to loading but also a chronic adaptation of the musculoskeletal system. We hypothesize that higher resting decorin levels may be indicative of a well-remodeled, mechanically robust ECM capable of superior elastic energy storage and return. If confirmed, this association would suggest that decorin may reflect musculoskeletal factors influencing running economy. However, given the cross-sectional design of this study, these findings indicate an association rather than a causal link.

#### 4.1.2. Hypoxanthine as an Individualized Marker of Maximal Metabolic Capacity

Higher post-test hypoxanthine was correlated significantly with faster 2.4 km Cooper test times, but not with 10 km race performance. This suggests that post-test hypoxanthine could reflect the acute metabolic strain of near-maximal exertion, rather than the metabolic efficiency required for submaximal endurance. This aligns with research showing that anaerobic training enhances the purine nucleotide cycle to manage rapid ATP turnover, framing a large post-exercise hypoxanthine response as a marker of metabolic power, not inefficiency [44,45].

This dynamic was evident when comparing training status. HTS runners, capable of sustaining a higher absolute work rate, exhibited a greater exercise-induced increase in hypoxanthine, reflecting acute ATP degradation that outpaced regeneration and salvage pathways [21]. Conversely, LTS runners showed higher baseline hypoxanthine but a smaller exercise-induced change—a pattern previously associated with reduced metabolic efficiency and impaired purine salvage [23]. While seemingly contrary to findings in elite cohorts, where lower post-exercise hypoxanthine indicates long-term adaptation [46], our results are consistent with the principle that near-maximal efforts in well-trained individuals provoke a significant accumulation of hypoxanthine [21]. This could also explain why post-test hypoxanthine did not predict performance in the submaximal 10 km race, where purine salvage and efficiency are more critical than maximal metabolic power.

#### 4.1.3. NT-proBNP as a Marker Defining the Threshold of Cardiac Stress

An important observation was the lack of a significant change in post-test NT-proBNP, despite near-maximal exertion. While prolonged endurance events like marathons consistently elicit post-exercise increases in NT-proBNP due to sustained ventricular wall stretching [47], the levels in trained individuals rarely exceed clinical cut-offs [48]. In contrast, maximal treadmill protocols, which involve a progressive workload until failure, can induce transient NT-proBNP increases, particularly in untrained individuals [28]. The null result for NT-proBNP may stem from a kinetic mismatch; the relatively short duration of the 2.4 km test may not provide the sustained cardiac wall stress required for significant peptide accumulation, rendering it insensitive for predicting performance in this specific protocol.

#### 4.1.4. BDNF as an Indicator of Exertion Rather than Performance Capacity

Although post-test BDNF increased significantly, it did not correlate with performance in the cohort as a whole, suggesting that it could reflect the acute neurobiological exertion rather than differentiating endurance capacity, likely explaining its physiological irrelevance to the 10 km race time prediction model. This aligns with current research showing an intensity-dependent regulation of BDNF [49,50].

When stratified by training status, baseline BDNF in HTS runners was positively correlated with race times. Although seemingly paradoxical given BDNF’s neuroprotective role, this finding aligns with data showing that higher $\overset{˙}{V}$O_2_max is associated with lower basal BNDF levels [27]. It could be hypothesized that this is a positive adaptation mediated by enhanced receptor sensitivity or more efficient uptake by the central nervous system. Alternatively, elevated baseline BDNF could reflect accumulated training stress, neuroinflammation, or insufficient recovery, although we acknowledge that this interpretation is speculative. Conversely, LTS runners exhibited higher baseline BDNF with no link to performance—a pattern seen in sedentary populations [51]. While post-test BDNF increased in both groups, the magnitude of the increase was lower in LTS runners and was associated with metabolic stress markers (LDH). This reinforces the interpretation that the acute BDNF response is a universal, threshold-based neurobiological reaction to high-intensity exertion, serving as a marker of relative effort rather than performance potential [26,27].

### 4.2. Race Time Prediction Model

Combining post-test decorin with BMI and Riegel-predicted race time offered a minor improvement for 10 km race time prediction in this sample. This is in line with the multidimensional view where aerobic capacity, while fundamental, is modulated by other physiological domains to determine performance [12,52]. The approach aligns with the trend toward using advanced statistical models, including regression shrinkage methods like Ridge and LASSO [53], to uncover patterns missed by conventional methods [54]. A biomarker could provide complementary information on a factor like running economy, which can vary by up to 30% among athletes with similar aerobic capacity [55]. In this instance, post-test decorin may have reflected musculoskeletal robustness, helping to better explain how athletes perform than standalone aerobic fitness.

In this regard, it is important to consider the mechanistic role of decorin in the context of field-based prediction errors. Both the Riegel formula and estimated $\overset{˙}{V}$O_2_max are derived from run velocity. Furthermore, $\overset{˙}{V}$O_2_max estimation penalizes body mass without distinguishing between functional contractile tissue and non-functional adipose tissue. Since decorin correlates with muscle mass, its inclusion in the model could serve as a biological ‘correction factor.’ Unlike descriptive studies that focus on marker changes, this proof of concept indicates that decorin could act complementary, rather than redundant, to 2.4 km Cooper-derived $\overset{˙}{V}$O_2_max. This could be due to capturing unique musculoskeletal information that improves prediction error even after accounting for velocity-based estimates. Consequently, the predictive value of decorin might be attenuated in settings where $\overset{˙}{V}$O_2_max is directly measured via gas analysis, although its utility for field-based screening remains robust.

Although the improvement was minor, decorin’s added value was consistent across different analytical approaches (Ridge and Elastic Net regression; in-sample and LOOCV evaluation). This consistency reinforces the view that a multidimensional assessment provides a more robust picture of endurance performance than aerobic capacity alone. Ultimately, this highlights the distinction between mechanistic relevance—where three of the four markers changed—and predictive utility, where only decorin provided stable incremental value. However, it is important to make a distinction between the statistical and practical significance of the incremental performance prediction improvement. In this regard, it must be noted that the reduction in prediction error is within normal day-to-day variation in 10 km race performance for recreational runners.

### 4.3. Limitations

Our cohort’s heterogeneity in sex and training background enhanced ecological validity of our findings for a broad recreational running population but also introduced variability that may limit model precision. This design choice was deliberate as our goal was to develop a proof of concept applicable across a heterogeneous recreational running population, rather than a narrowly optimized model for a specific demographic. Due to the specific characteristics of the study population (recreational and club-level runners) and environmental context (temperate continental climate), our findings may not directly transfer to elite cohorts or different environmental extremes. Furthermore, the use of an official 10 km road race as the criterion measure introduced uncontrolled variables inherent to mass-participation events. However, this design choice was intentional to prioritize ecological validity, ensuring that the prediction model reflects performance in the actual competitive environment.

The reported regression coefficients should be viewed as preliminary, due to limited statistical power. As the a priori power analysis was calculated for detecting acute biomarker responses, the sample size for the predictive model yields an events-per-variable ratio lower than traditional recommendations for standard multiple linear regression. To mitigate the risk of overfitting, we employed Ridge regression, which is specifically designed for such scenarios. Ridge introduces a penalty term to shrink coefficient estimates, thereby reducing model variance and improving generalizability even when sample sizes are limited [38].

The use of estimated $\overset{˙}{V}$O_2_max means that the predictive improvement from decorin might partially reflect a correction of the estimation error inherent in the formula. Future studies using directly measured $\overset{˙}{V}$O_2_max are needed to disentangle whether decorin provides independent predictive information.

The 10 min window for post-exercise blood sampling, while practical, may have introduced measurement error due to the rapid kinetics of hypoxanthine and interindividual differences in its peak timing [44], potentially attenuating the observed correlations. Furthermore, while the sampling window was favorable for capturing large shifts in hypoxanthine, decorin and BDNF due to their rapid increase, the slower kinetics of NT-proBNP release coupled with a 2.4 km Cooper test duration might have contributed to its lack of significance [17,23,27,28]. Future studies utilizing multiple post-exercise time points could further elucidate the relationship between biomarker clearance rates and performance capacity.

### 4.4. Practical Applications

While this proof of concept indicates that decorin could offer incremental improvement for performance prediction, the reduction in prediction error was within normal day-to-day variation for recreational runners. Given the practical costs of ELISA-based decorin quantification and the logistical requirements of venous blood sampling, this benefit does not justify routine implementation for individual athlete monitoring. Instead, it might be informative when applied strategically—specifically, at the conclusion of high-volume base training blocks to support musculoskeletal adaptation. The greatest potential may, therefore, reside in longitudinal monitoring, as it could potentially help identify adaptation plateaus or early signs of non-functional overreaching. Since we have demonstrated that the 2.4 km Cooper test elicits measurable biomarker kinetics, it may serve as a logical, ecologically valid standardized stressor for longitudinal monitoring.

### 4.5. Future Research

Future studies should employ longitudinal designs to track biomarker changes alongside performance improvements over a full training season, as well as examine whether sex-stratified or training-status-specific models provide superior predictive accuracy. External validation in larger, homogeneous cohorts is essential to confirm decorin’s utility. Furthermore, comparing these models against directly measured $\overset{˙}{V}$O_2_max would clarify whether decorin provides truly independent information or merely corrects the errors inherent in estimated aerobic capacity.

## 5. Conclusions

This proof-of-concept study provides preliminary evidence that post-test decorin, when added to conventional field-based predictors, provides a consistent—though practically minor—incremental improvement in 10 km race time prediction.

While the absolute reduction in prediction error is small, these findings suggest that post-test decorin might offer insight into musculoskeletal robustness—not fully reflected by velocity-based field tests like 2.4 km Cooper. Thus, post-test decorin may be a candidate for enhancing the accuracy of performance prediction models rather than directly reflecting performance capacity itself. To transition from this proof of concept to practical application, future research must employ longitudinal designs to track biomarker dynamics across training cycles, utilize external validation in larger, homogeneous cohorts to minimize physiological noise, and compare these results against gold-standard, laboratory-measured $\overset{˙}{V}$O_2_max.

## Figures and Tables

**Table 1:** Demographic, anthropometric, and body composition characteristics of the study population (*n* = 33).

Variable		Descriptives	(BCa) 95% CI
Sex			
	Male	22 (66.7%)	
	Female	11 (33.3%)	
Age (year)		25 (20–29)	[23,24,25,26]
Body Mass (kg)		65.4 (60.0–75.7)	[61.1–70.5]
Height (m)		1.77 (1.73–1.82)	[1.76–1.79]
BMI (kg m^−2^)		20.9 (19.6–23.0)	[20.0–21.8]
Body Fat (%)		14.1 (10.2–16.5)	[11.2–15.6]
Fat Mass (kg)		8.9 (6.6–11.6)	[7.6–10.8]
Muscle Mass (kg)		54.4 (48.9–63.3)	[51.4–59.9]

All continuous variables are presented as the median (25th–75th percentile) with bias-corrected and accelerated (BCa) bootstrap 95% confidence intervals (CIs; 1000 resamples). Most variables did not follow a normal distribution (Shapiro–Wilk test, *p* ≤ 0.05); therefore, a consistent non-parametric summary was used. The categorical variable (sex) is presented as counts and percentages (*n*, %). BMI—body mass index.

**Table 2:** Running performance metrics of the study population (*n* = 33).

Variable	Descriptives	95% CI
2.4 km Cooper Test Time (min)	9.33 ± 1.66	[8.74–9.92]
Estimated $\overset{˙}{V}$O_2_max (mL kg^−1^ min^−1^) ^a^	53.37 ± 5.76	[51.33–55.41]
Predicted 10 km Race Time (min) ^b^	42.34 ± 7.53	[39.67–45.01]
Observed 10 km Race Time (min) ^c^	43.19 ± 8.19	[39.73–46.65]

Values are presented as the mean ± standard deviation (*SD*) with 95% confidence intervals (CIs), as variables followed a normal distribution (Shapiro–Wilk test, *p* > 0.05). ^a^ Estimated $\overset{˙}{V}$O_2_max represents maximal oxygen uptake derived from the 2.4 km Cooper test performance. ^b^ Predicted 10 km race time was derived from the 2.4 km Cooper test using the Riegel formula. ^c^ Data available for 24 participants.

**Table 3:** Sequential improvement in 10 km race time prediction using Ridge regression.

Model	Predictors Included	In-Sample				LOOCV		
		*R* ^2^	MAE (min)	RMSE (min)		*R* ^2^	MAE (min)	RMSE (min)
1	Riegel-Predicted 10 km Race Time (min)	0.927	1.76	2.17		0.912	1.92	2.38
2	Riegel-Predicted 10 km Race Time (min) + BMI (kg m^−2^)	0.943	1.56	1.91		0.927	1.77	2.17
3	Riegel-Predicted 10 km Race Time (min) + BMI (kg m^−2^) + decorin_post_ (ng mL^−1^)	0.955	1.37	1.70		0.933	1.69	2.08

In-sample—performance evaluated on the same data used to train the model; LOOCV (leave-one-out cross-validation)—providing an internally cross-validated estimate of predictive performance. In-sample *R*^2^ reflects model fit, whereas LOOCV metrics (including cross-validated *R*^2^) reflect predictive performance. The regularization parameter *α* was selected by 6-fold cross-validation for in-sample models (*α* = 0.01 for all models) and re-tuned within each LOOCV iteration using nested 5-fold cross-validation. Post—biomarker concentrations measured after completion of the 2.4 km Cooper test; BMI—body mass index; *R*^2^—coefficient of determination; MAE—mean absolute error; RMSE—root-mean-square error.

**Table 4:** Standardized and unstandardized coefficients with bias-corrected and accelerated (BCa) bootstrap 95% confidence intervals (CIs) for the three-predictor 10 km race time prediction model.

Variable	Standardized Coefficients [BCa 95% CI]	Unstandardized Coefficients [BCa 95% CI]
Intercept	43.187 [40.136, 46.477]	−7.860 [−16.182, 1.367]
Riegel-Predicted 10 km Race Time (min)	6.594 [4.875, 8.763]	0.971 [0.787, 1.156]
BMI (kg m^−2^)	1.783 [0.609, 3.523]	0.704 [0.251, 1.179]
Decorin_post_ (ng mL^−1^)	−1.289 [−2.520, −0.063]	−0.761 [−1.430, 0.041]

BMI—body mass index; post—biomarker concentrations measured after completion of the 2.4 km Cooper test.

## Data Availability

The original contributions presented in the study are included in the article/Supplementary Material, and further inquiries can be directed to the corresponding author.

## Supplementary Materials

The following supporting information can be downloaded at: https://www.mdpi.com/article/10.3390/life16020320/s1, Table S1: Analytical characteristics of biomarker assay kits; Table S2: Demographic, anthropometric, and body composition characteristics by sex; Table S3: Demographic, anthropometric, and body composition characteristics by training status; Table S4: Concentrations of conventional blood-based markers following the 2.4 km Cooper test in the study population (*n* = 33); Figure S1: Blood-based biomarker changes after the 2.4 km Cooper test by sex and training status; Figure S2: Correlations between blood-based biomarkers and 2.4 km Cooper test running time by training status; Figure S3: Correlations between blood-based biomarkers and 10 km road race time by training status; Figure S4: Correlations between non-conventional and conventional markers by training status; Figure S5: Observed versus predicted 10 km race time for the final three-predictor Ridge regression model.

## Abbreviations

The following abbreviations are used in this manuscript:

NT-proBNP	N-terminal pro-B-type natriuretic peptide
BDNF	Brain-derived neurotrophic factor
CK	Creatine kinase
LDH	Lactate dehydrogenase
TCR	Testosterone-to-cortisol ratio
NLR	Neutrophil-to-lymphocyte ratio
ATP	Adenosine triphosphate
ECM	Extracellular matrix
$\overset{˙}{V}$O_2_max	Maximal oxygen uptake
BMI	Body mass index
HTS	Higher training status
LTS	Lower training status
