# Supplementary material for: Can Non-Conventional Blood Biomarkers Improve Running Performance Prediction? A Proof of Concept
Source: Life (Basel). 2026 Feb 12;16(2):320. doi: 10.3390/life16020320 (PMC12941692; doi:10.3390/life16020320)
Supplement: Supplementary file 1 [file life-16-00320-s001.zip › life-4139872-supplementary.pdf]

Supplementary Materials: Table S1. Analytical characteristics of biomarker assay kits; Table S2: Demographic, anthropometric, and body composition characteristics by sex; Table S3: Demographic, anthropometric, and body composition characteristics by training status; Table S4: Concentrations of conventional blood-based markers following the 2.4 km Cooper test in the study population ($n = 33$); Figure S1: Blood-based biomarker changes after the 2.4 km Cooper test by sex and training status; Figure S2: Correlations between blood-based biomarkers and 2.4 km Cooper test running time by training status; Figure S3: Correlations between blood-based biomarkers and 10 km road race time by training status; Figure S4: Correlations between non-conventional and conventional markers by training status; Figure S5: Observed versus predicted 10 km race time for the final three-predictor Ridge regression model.

Table S1. Analytical characteristics of biomarker assay kits.

Biomarker	Assay kit	Intra-assay CV	Inter-assay CV	MDD
Decorin	Abcam Human Decorin ELISA (ab99998)	<10%	<12%	<1.5 pg mL^{-1}
Hypoxanthine	Abcam Xanthine/Hypoxanthine Assay Kit (ab155900)	–	–	0.4 μmol L^{-1}
NT-proBNP	Abcam Human NT-proBNP ELISA (ab263877)	4.3%	5.6%	11.5 pg mL^{-1}
BDNF	Abcam Human BDNF ELISA (ab212166)	2.8%	5.3%	1.5 pg mL^{-1}

NT-proBNP—N-terminal pro-B-type natriuretic peptide; BDNF—brain-derived neurotrophic factor; ELISA—enzyme-linked immunosorbent assay; CV—coefficient of variation; MDD—minimum detectable dose

Table S2. Demographic, anthropometric, and body composition characteristics by sex.

Variable	Male ($n = 22$)	Female ($n = 11$)	p
Age (year)	23 (19–27) [20–26]	27 (23–31) [24–29]	0.143
Body mass (kg)	70.6 (62.8–79.8) [65.4–78.2]	57.6 (54.3–64.4) [56.7–62.0]	<0.001
Height (m)	1.81 (1.77–1.88) [1.78–1.84]	1.72 (1.68–1.74) [1.69–1.73]	<0.001
BMI (kg m^{-2})	21.1 (20.0–24.1) [20.2–23.7]	20.0 (19.0–21.4) [19.4–21.1]	0.082
Body fat (%)	11.1 (7.2–14.5) [9.3–12.3]	19.4 (15.9–21.2) [16.4–20.3]	<0.001
Fat mass (kg)	7.7 (5.2–10.7) [5.9–10.0]	10.8 (8.9–12.8) [9.2–12.0]	0.017
Muscle mass (kg)	60.6 (54.3–66.7) [56.6–64.2]	46.0 (43.1–49.2) [43.8–48.6]	<0.001

All continuous variables are presented as median (25th–75th percentile) with bias-corrected and accelerated (BCa) bootstrap 95% confidence intervals (CIs, 1000 resamples; square brackets). Most variables did not follow a normal distribution (Shapiro–Wilk test, $p \leq 0.05$), therefore a consistent

https://doi.org/10.3390/xxxxx

non-parametric summary was used. Between-group differences were assessed using the Mann–Whitney *U* test, with statistical significance set at $p < 0.05$. BMI—body mass index.

Table S3. Demographic, anthropometric, and body composition characteristics by training status

Variable	HTS (*n* = 19)	LTS (*n* = 14)	*p*
Age (year)	21 (19–26)<br>[19–24]	28 (25–31)<br>[26–30]	0.004
Body mass (kg)	63.4 (59.6–72.2)<br>[60.5–69.3]	69.5 (60.7–78.6)<br>[64.4–74.3]	0.397
Height (m)	1.78 (1.73–1.86)<br>[1.74–1.82]	1.76 (1.73–1.81)<br>[1.75–1.79]	0.439
BMI (kg m^{-2})	20.2 (19.0–21.4)<br>[19.7–20.9]	22.3 (20.6–24.8)<br>[21.0–23.8]	0.035
Body fat (%)	11.0 (6.7–14.10)<br>[9.8–12.3]	16.4 (14.5–21.1)<br>[14.5–21.1]	<0.001
Fat mass (kg)	7.5 (4.5–9.2)<br>[6.4–8.4]	11.7 (9.3–13.6)<br>[10.1–13.1]	<0.001
Muscle mass (kg)	54.4 (49.8–62.9)<br>[52.6–59.9]	55.4 (47.7–63.9)<br>[49.2–61.3]	0.760

All continuous variables are presented as median (25th–75th percentile) with bias-corrected and accelerated (BCa) bootstrap 95% confidence intervals (CIs, 1000 resamples; square brackets). Most variables did not follow a normal distribution (Shapiro–Wilk test, $p \leq 0.05$), therefore a consistent non-parametric summary was used. Between-group differences were assessed using the Mann–Whitney *U* test, with statistical significance set at $p < 0.05$ BMI—body mass index.

https://doi.org/10.3390/xxxxx

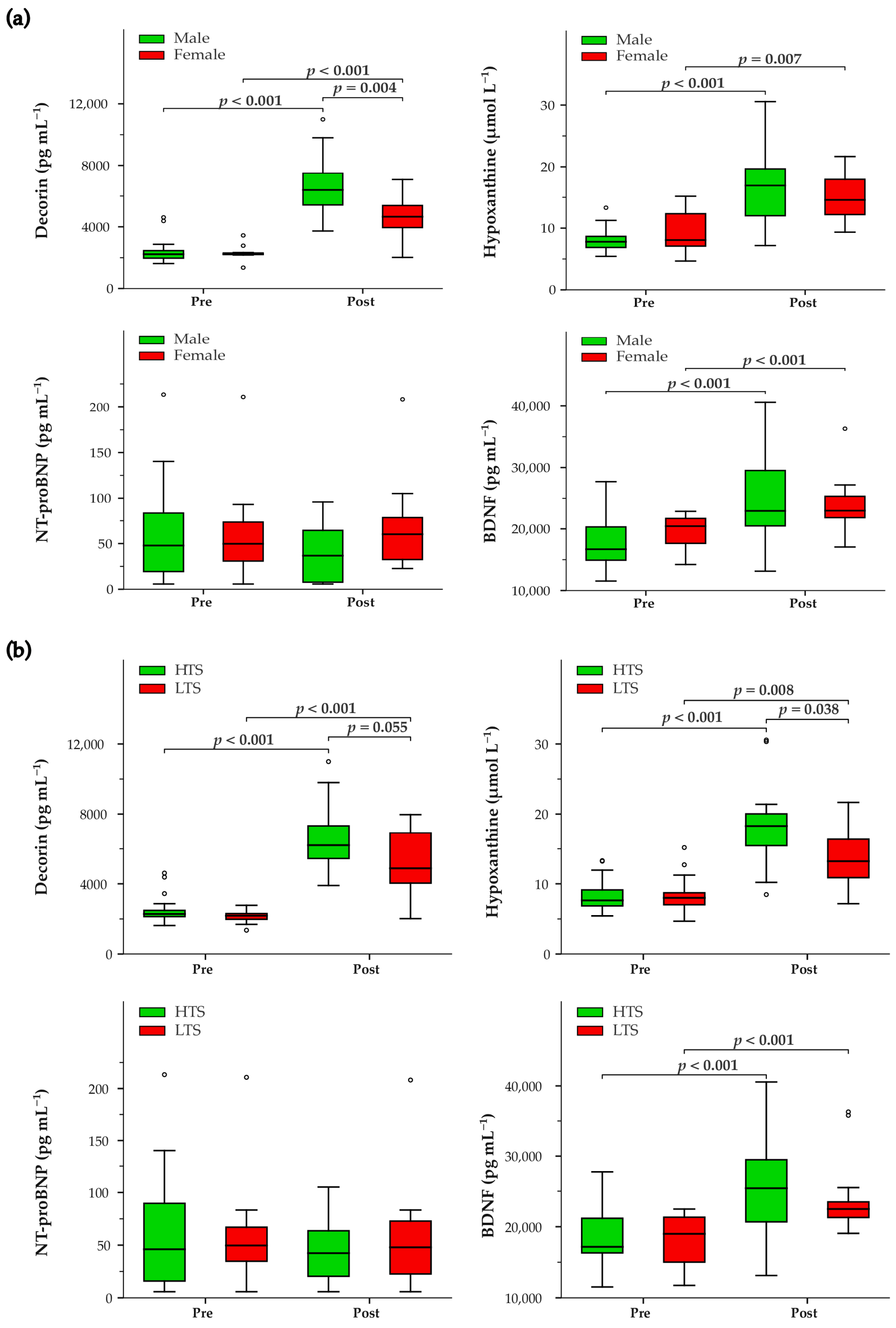

Figure S1. Blood-based biomarker changes after the 2.4 km Cooper test by sex and training status. Boxplots showing concentrations of decorin, hypoxanthine, NT-proBNP, and BDNF measured at baseline (Pre) and after (Post) the 2.4 km Cooper test, stratified by **(a)** sex (male, $n = 22$; female, $n = 11$) and **(b)** training status (HTS, $n = 19$; LTS, $n = 14$). Green boxes represent male or HTS participants, and red boxes represent female or LTS participants. Differences between Pre and Post within each group were tested using the Wilcoxon signed-rank test, while between-group comparisons (male vs. female, HTS vs. LTS) were assessed using the Mann–Whitney *U* test. Statistically significant differences ($p < 0.05$) are indicated above the boxes. The central line within each box indicates the median; whiskers denote 1.5 × interquartile range; circles indicate outliers. NT-proBNP—N-terminal pro-B-type natriuretic peptide; BDNF—brain-derived neurotrophic factor; HTS—higher training status; LTS—lower training status.

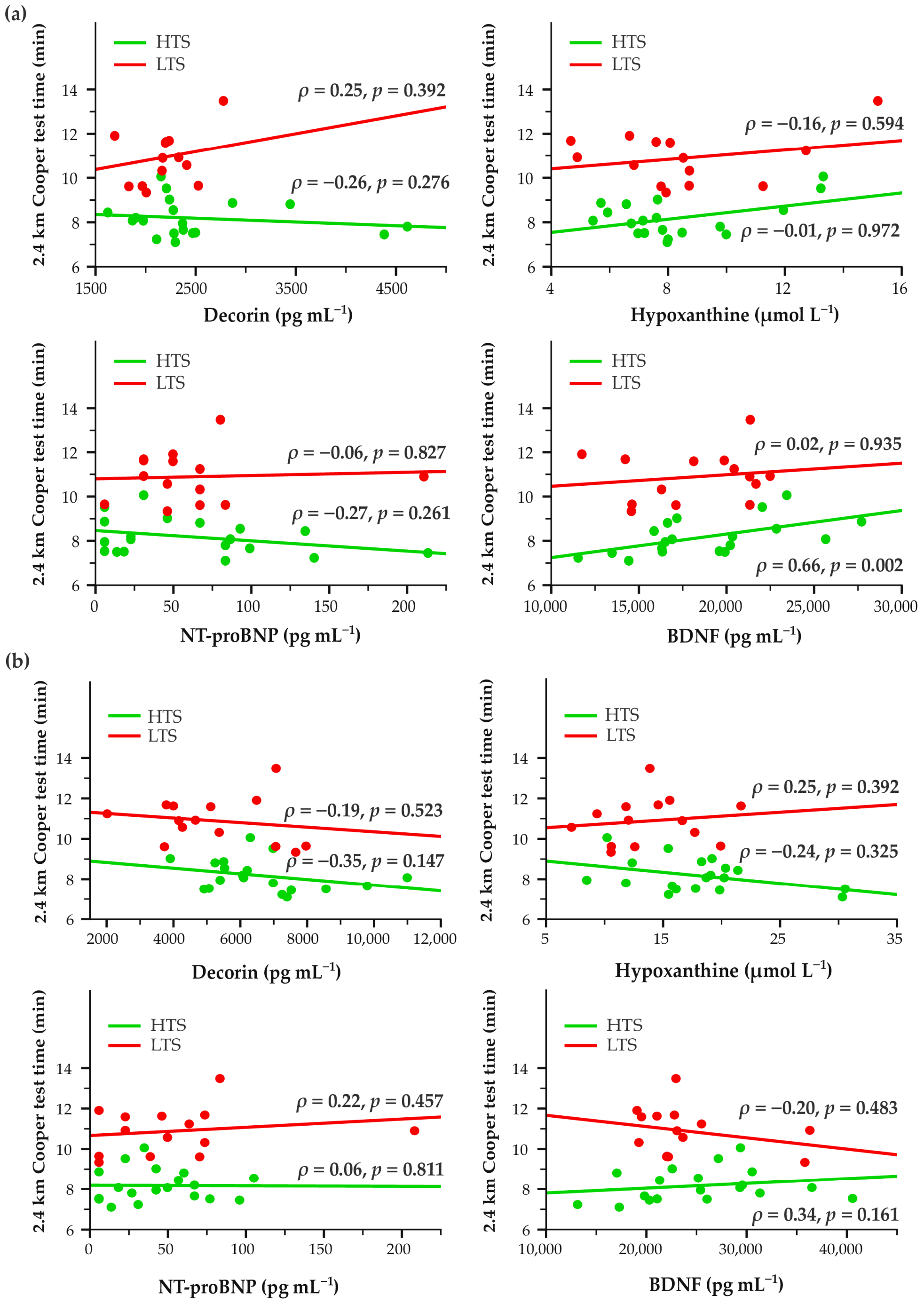

Figure S2. Correlations between blood-based biomarkers and 2.4 km Cooper test running time by training status. Scatterplots showing correlations between **(a)** baseline and **(b)** post-test biomarker concentrations and 2.4 km Cooper test running time, stratified by training status (HTS, $n = 19$; LTS, $n = 14$). Green points and lines represent HTS participants, and red points and lines represent LTS participants. Solid lines indicate linear trendlines. Correlation coefficients were computed using Spearman's ρ. Statistically significant correlation was determined at $p < 0.05$. NT-proBNP—N-terminal pro-B-type natriuretic peptide; BDNF—brain-derived neurotrophic factor; HTS—higher training status; LTS—lower training status.

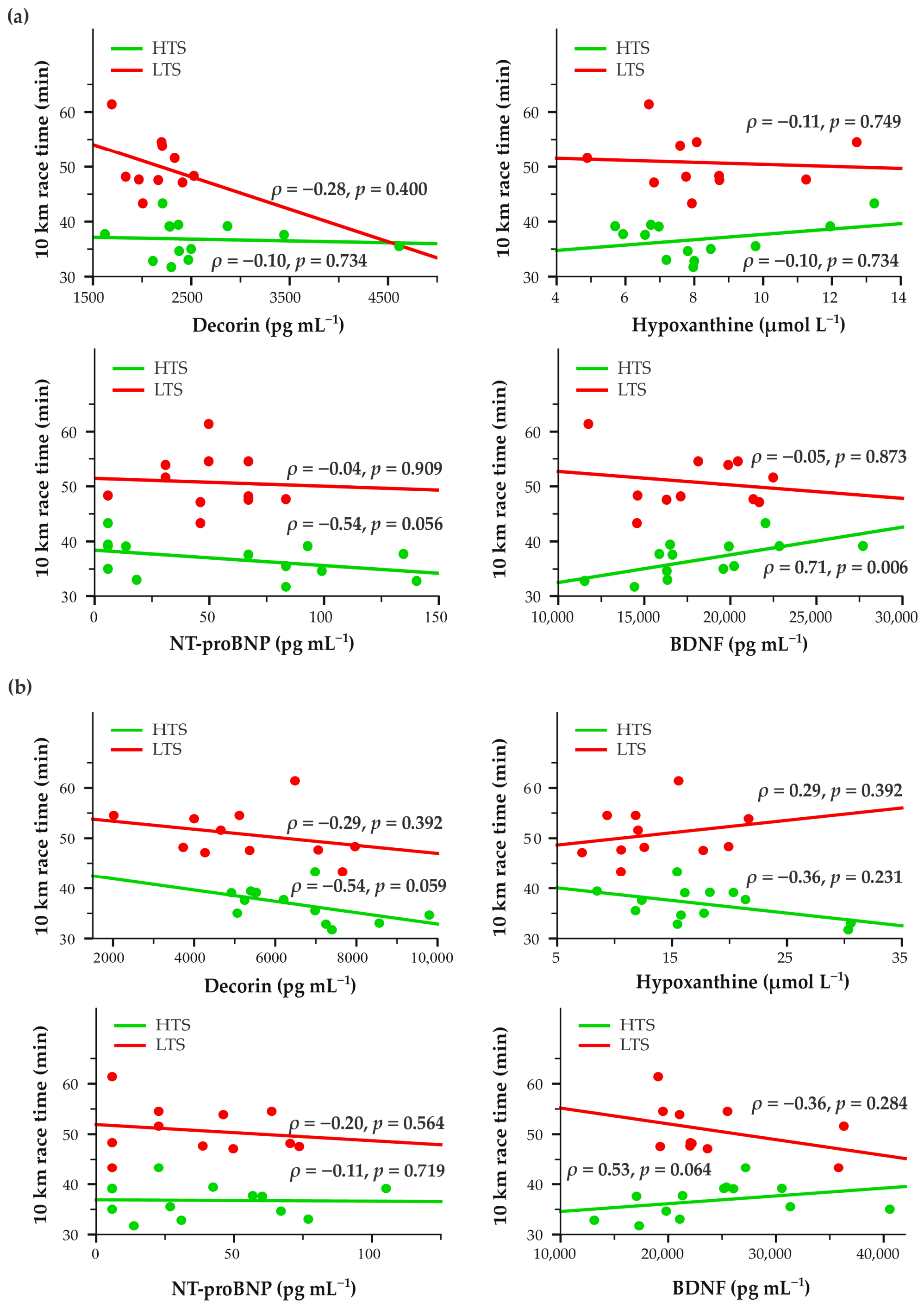

Figure S3. Correlations between blood-based biomarkers and 10 km road race time by training status. Scatterplots showing correlations between **(a)** baseline and **(b)** post-test biomarker concentrations (measured in relation to the 2.4 km Cooper test) and 10 km road race time, stratified by training status (HTS, $n = 13$; LTS, $n = 11$). Green points and lines represent HTS participants, and red points and lines represent LTS participants. Solid lines indicate linear trendlines. Correlation coefficients were computed using Spearman's ρ. Statistically significant correlation was determined at $p < 0.05$. NT-proBNP—N-terminal pro-B-type natriuretic peptide; BDNF—brain-derived neurotrophic factor; HTS—higher training status; LTS—lower training status.

Table S4. Concentrations of conventional blood-based markers following the 2.4 km Cooper test in the study population ($n = 33$).

Variable	Descriptives	(BCa) 95% CI
CK (U L^{-1})	245 (123–402)	[177–301]
LDH (U L^{-1})	215 (183–248)	[197–229]
Cortisol (nmol L^{-1})	424 (352–486)	[373–471]
Testosterone (nmol L^{-1})	18.8 (1.7–26.5)	[15.7–19.9]
Neutrophils (×10^9 L^{-1})	4.45 (3.85–5.43)	[4.34–4.77]
Lymphocytes (×10^9 L^{-1})	4.70 (4.16–5.81)	[4.52–5.30]
TCR	0.045 (0.004–0.064)	[0.043–0.050]
NLR	0.965 (0.773–1.073)	[0.887–1.000]

All continuous variables are presented as median (25th–75th percentile) with bias-corrected and accelerated (BCa) 95% bootstrap confidence intervals (CIs; 1000 resamples). Most variables did not follow a normal distribution (Shapiro–Wilk test, $p \leq 0.05$), therefore a consistent non-parametric summary was used. CK—creatine kinase; LDH—lactate dehydrogenase; TCR—testosterone-to-cortisol ratio; NLR—neutrophil-to-lymphocyte ratio.

https://doi.org/10.3390/xxxxx

(a)

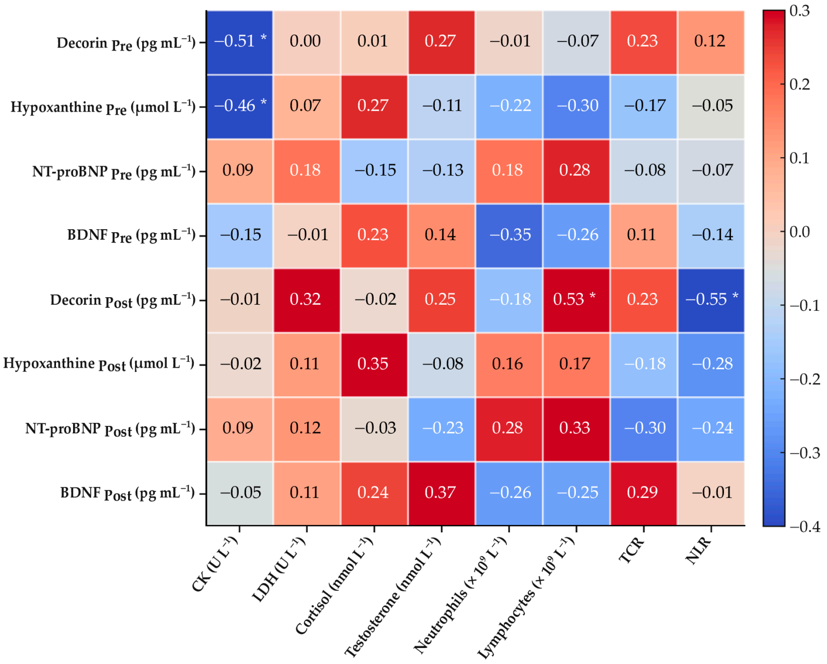

(b)

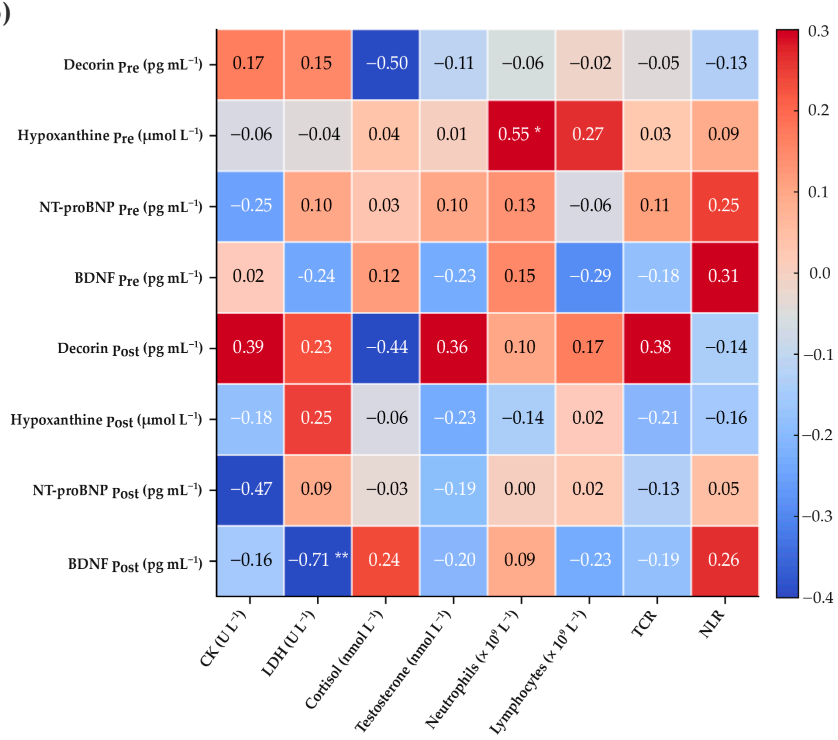

Figure S4. Correlations between non-conventional and conventional markers by training status. Heatmaps showing correlations between non-conventional blood-based biomarkers (decorin, hypoxanthine, NT-proBNP, and BDNF) measured at baseline (Pre) and after (Post) the 2.4 km Cooper test, and conventional markers measured after the test in **(a)** HTS (higher training status, $n = 19$) and (b) LTS (lower training status, $n = 14$) participants. Numbers represent correlation coefficients computed using Spearman's ρ. Asterisks indicate significant correlations (*$p < 0.05$, **$p < 0.01$). NT-proBNP—N-terminal pro-B-type natriuretic peptide; BDNF—brain-derived neurotrophic factor; CK—creatine kinase; LDH—lactate dehydrogenase; TCR—testosterone-to-cortisol ratio; NLR—neutrophil-to-lymphocyte ratio.

https://doi.org/10.3390/xxxxx

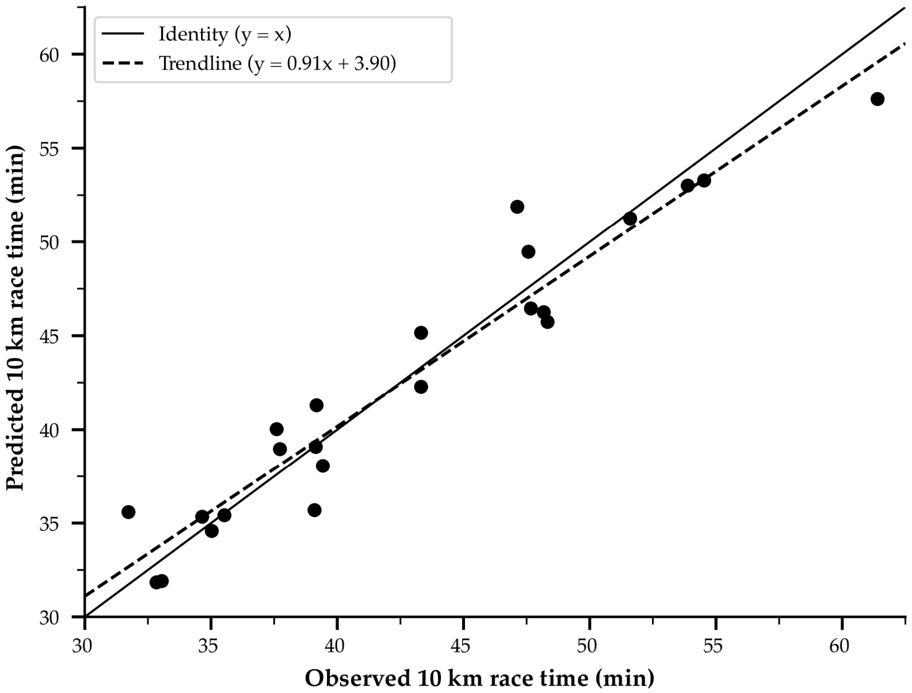

Figure S5. Observed versus predicted 10 km race time for the final three-predictor Ridge regression model. Predicted values were obtained using leave-one-out cross-validation. The solid line represents the line of identity (y = x), and the dashed line represents the fitted linear trend.

https://doi.org/10.3390/xxxxx
